# The geographic distribution of onchocerciasis in the 20 participating countries of the African Programme for Onchocerciasis Control: (1) priority areas for ivermectin treatment

**DOI:** 10.1186/1756-3305-7-325

**Published:** 2014-07-22

**Authors:** Mounkaila Noma, Honorat GM Zouré, Afework H Tekle, Peter AI Enyong, Bertram EB Nwoke, Jan HF Remme

**Affiliations:** 1 African Programme for Onchocerciasis Control, BP 549 Ouagadougou, Burkina Faso; 2 Tropical Medicine Research Station, Kumba, Cameroon; 3 Public Health Parasitology & Entomology Unit, Imo State University, Owerri, Nigeria; 4 120 Rue des Campanules, 01210 Ornex, France

**Keywords:** Onchocerciasis, APOC, Onchocercal nodule, Mapping, REMO, High risk areas, Ivermectin, Community-directed treatment

## Abstract

**Background:**

The African Programme for Onchocerciasis Control (APOC) was created to control onchocerciasis as a public health problem in 20 African countries. Its main strategy is community directed treatment with ivermectin. In order to identify all high risk areas where ivermectin treatment was needed, APOC used Rapid Epidemiological Mapping of Onchocerciasis (REMO). REMO has now been virtually completed and we report the results in two articles. The present article reports the mapping of high risk areas where onchocerciasis was a public health problem. The companion article reports the results of a geostatistical analysis of the REMO data to map endemicity levels and estimate the number infected.

**Methods:**

REMO consists of three stages: exclusion of areas that are unsuitable for the vector, selection of sample villages to be surveyed in each river basin, and examination of 30 to 50 adults for the presence of palpable onchocercal nodules in each selected village. The survey results and other relevant information were processed in a geographical information system. A panel of experts interpreted the data taking the river-based sampling into account and delineated high risk areas where the prevalence of nodules is greater than 20%.

**Results:**

Unsuitable areas were identified in eight countries. In the remaining areas surveys were done in a total of 14,473 sample villages in which more than half a million people were examined. High-risk areas were identified in 18 APOC countries, ranging from small isolated foci to a vast contiguous endemic area of 2 million km^2^ running across seven countries. In five countries the high risk area covered more than 48% of the total surface area, and 31% to 48% of the population. It is estimated that 86 million people live in high risk areas in the APOC countries.

**Conclusions:**

The REMO maps have played a significant role in onchocerciasis control in the 20 APOC countries. All high-risk areas where onchocerciasis used to be a serious public health problem have been clearly delineated. This led to the creation of community-directed treatment projects that by 2012 were providing annual ivermectin treatment to over 80 million people.

## Background

**Figure 1:**
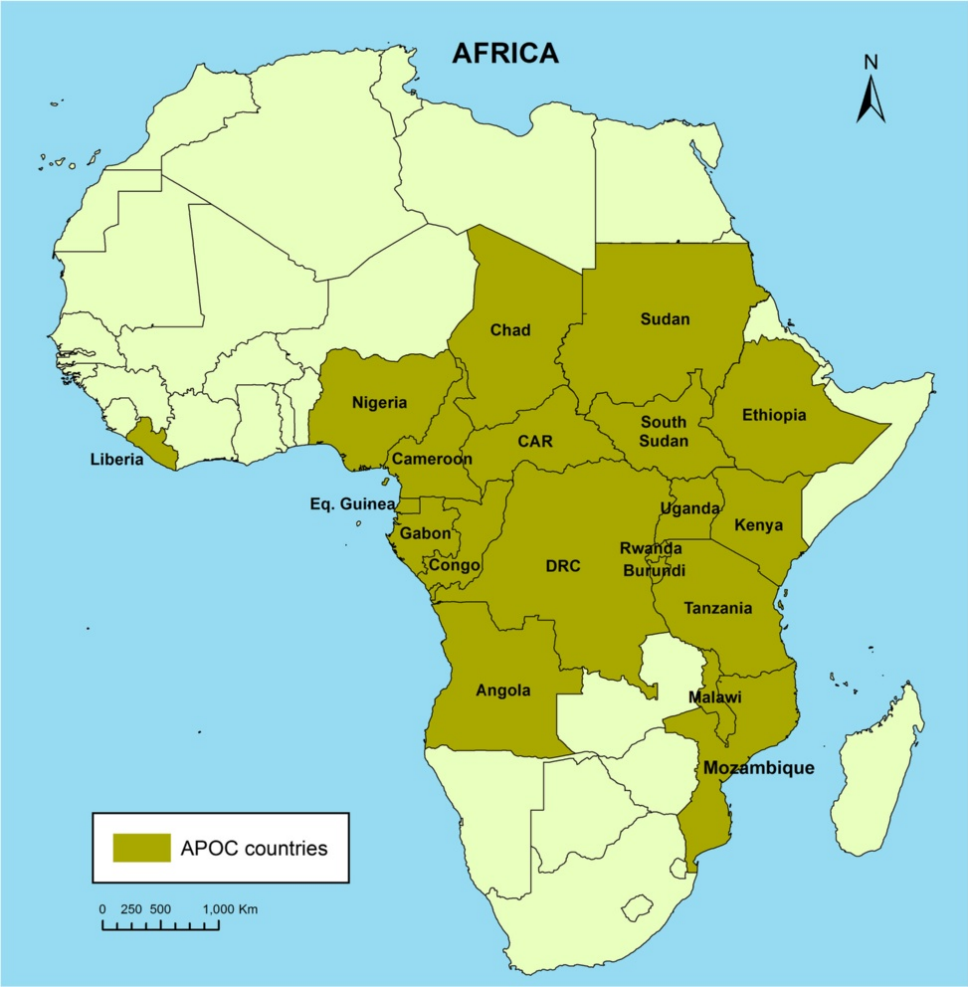
Participating countries in the African Programme for Onchocerciasis Control (APOC).

Onchocerciasis, or river blindness, is caused by infection by the filarial worm *Onchocerca volvulus* which is transmitted by female black flies of the genus *Simulium*. Onchocerciasis used to be endemic in some 30 countries in Africa where over 99% of all cases in the world were found [1]. In most of these countries, onchocerciasis was a severe public health problem, responsible for blindness and visual impairment, debilitating skin disease and relentless itching in millions of people, and the disease had serious socio-economic consequences, including depopulation of fertile river valleys and reduced productivity of affected persons [1-3]. Since 1975 there have been large-scale efforts to control the disease as both a public health problem and obstacle to socio-economic development. The Onchocerciasis Control Programme in West Africa (OCP) has successfully controlled onchocerciasis by vector control in the savanna belt of nine West African countries [4]. However, in the remaining endemic areas in Africa, where the large majority of onchocerciasis cases lived, vector control was not considered feasible or cost-effective and no chemotherapy existed that was suitable for onchocerciasis control [5]. The registration of ivermectin for the treatment of human onchocerciasis in 1987 was therefore a major breakthrough. The evidence from community trials that annual ivermectin treatment could effectively control the disease and the commitment by the manufacturer to donate ivermectin free of charge for as long as needed led to a rapid expansion of onchocerciasis control activities [6,7]. An international coalition of Non-Governmental Development Organizations (NGDOs) spearheaded ivermectin distribution efforts [8] and in 1995 the African Programme for Onchocerciasis Control (APOC; see Figure 1) was created with the mandate to support the establishment of community directed treatment with ivermectin (CDTi) in all remaining areas in Africa where onchocerciasis was a public health problem [5].

One of the first challenges for APOC was to determine exactly where onchocerciasis was a public health problem and CDTi was a priority. Historical information did exist on the distribution of onchocerciasis in the 20 APOC countries in the form of reports on prevalence surveys in single or clusters of villages [9-30], reviews of the available information on the distribution of onchocerciasis in different countries [31-56] and attempts by WHO to interpret all this information and draw maps of the approximate distribution of onchocerciasis in Africa [1,57,58]. However, for most areas the available information was either incomplete or not accurate and reliable enough for targeting ivermectin treatment programmes [5,59]. There was, therefore, an urgent need for comprehensive mapping of the geographic distribution of onchocerciasis in all potentially endemic countries in Africa outside the OCP [60]. This was a vast area of some 14 million km^2^ and it would have been extremely difficult to determine the distribution of onchocerciasis throughout this area using the mapping and survey methods that were current at that time [61]. The distribution of different vector species and the location of the breeding sites were not known, while the principal diagnostic tool in use was a parasitological method based on the microscopic examination of skin biopsies; a time-consuming and invasive method. In response to these problems, a rapid assessment method for the Rapid Epidemiological Mapping of Onchocerciasis (REMO) was developed in 1993 and successfully tested at scale in Cameroon and Nigeria [62].

The geographic distribution of onchocerciasis is largely determined by the ecology and behavior of the vector [63]. Simulium vector flies breed in fast flowing, well oxygenated rivers and streams with adequate nutrients. The adult female flies disperse mainly along the river and their flight range rarely exceeds more than 15 km away from the river when seeking a blood meal [63-65]. Hence, the highest prevalence rates of onchocerciasis infection are almost invariably observed among villages located close to rivers with *Simulium* breeding sites. REMO uses this knowledge to identify potentially endemic areas and to select sample villages to be surveyed taking the ecology and the spatial behaviour of Simulium vectors into account.

The selected sample villages are surveyed using a non-invasive rapid assessment method to estimate their level of onchocerciasis endemicity. In each selected village a sample of adults is examined for the presence of palpable subcutaneous onchocercal nodules and the prevalence of nodules is calculated. Previous studies had shown that the severity of onchocercal disease in the community, and thus its public health importance, is related to the level of onchocerciasis endemicity as also reflected in the prevalence of nodules [2,66,67]. Based on available data on this relationship, an expert committee recommended in the early 1990s that, in order to control onchocerciasis as a public health problem, ivermectin treatment should be provided to all high risk communities where the prevalence of onchocercal nodules in adults was greater than 20% [68].

The development of REMO came just in time for APOC, which swiftly adopted this rapid assessment method and supported its large-scale application for mapping onchocerciasis in all APOC countries in order to identify priority areas for CDTi [59]. REMO thus became an essential first step in the planning and implementation of national onchocerciasis control programs supported by APOC. Large scale application of REMO started in 1996, and has since been applied in phase with the expansion of CDTi to cover all potentially endemic areas in APOC countries.

To date, virtually all potentially endemic areas in the 20 APOC countries have been mapped for onchocerciasis through REMO. Some partial results have been reported previously [59,69-73]. We report the complete results of the REMO surveys and the spatial analysis of the REMO data in two articles. The present article summarises the REMO surveys and shows the results of an expert analysis to delineate high-risk areas where onchocerciasis was a major public health problem and where ivermectin treatment was a priority for onchocerciasis control. These REMO maps of high risk areas have been the basis for the delineation of the CDTi projects that by 2011 were treating over 80 million people in the APOC countries [74]. In a companion paper we report the results of a geostatistical analysis of the REMO data using a methodology that was recently introduced in APOC [75] and that has allowed the mapping of onchocerciasis endemicity levels and the estimation of the number of people that would have been infected in the absence of control [76].

## Methods

### REMO methodology

The spatial epidemiology of onchocerciasis, or river blindness, is closely related to the distribution of local river systems, their suitability for simulium breeding and the flight range of the vector when seeking a blood meal. REMO is based on this knowledge and consists of three stages [77]:

1) The division of the area that needs to be mapped into biogeographic zones that are reasonably uniform with regard to their potential for onchocerciasis and that cover the watersheds of the main local drainage systems. Areas that are known to be unsuitable for the vector for ecological reasons (absence of fast flowing water, high altitude, etc) and uninhabited national parks are excluded at this stage.

2) The selection of a sample of villages to be surveyed in order to determine whether onchocerciasis is present or not and, if present, to give a rough indication of the distribution and severity of onchocerciasis in the zone. This sampling uses the available information on the local river system and involves two steps for each river basin:

a) Selection of villages at high risk locations. These are villages that are located in places where the risk of onchocerciasis is likely to be highest, i.e. close to the river bank, close to rapids and without other human settlements between them and the river. At least one high risk village is to be sampled every 30 to 50 km along the river and each major tributary.

b) Selection of secondary villages. For each high risk village, a related secondary village is selected which is located at least 10 km away from the river and the likely source of vectors.

3) Rapid epidemiological assessment (REA) surveys in the selected villages. A sample of 30-50 adults aged 20 years or more who are resident in the village for at least 10 years are examined for the presence of nodules, and the percentage of adults with palpable onchocercal nodules is calculated. The geographic coordinates of each village are collected using a hand-held Global Positioning System (GPS) receiver in a central location in the village.

The full details of the REMO methodology are provided in the WHO Manual for Rapid Epidemiological Mapping of Onchocerciasis [77].

### REMO implementation in APOC countries

The implementation of REMO was the responsibility of the Ministry of Health of each APOC country in collaboration with its partners in onchocerciasis control and with technical and financial support from APOC [59]. Each APOC country has a National Onchocerciasis Task Force (NOTF) that brings the various partners together in order to coordinate the onchocerciasis control activities in the country. The manager of the NOTF is the responsible officer for onchocerciasis control in the Ministry of Health. The NOTF of each country submitted to APOC a plan of action and budget for REMO. Upon approval this plan, APOC provided the agreed funding and external experts to train and support national teams with the implementation of REMO. Some REMO activities were funded and/or undertaken by NGDOs, but always under the auspices of the Ministry of Health and following the WHO guidelines for REMO surveys.

During the first years of APOC, priority for REMO was given to those areas where local partners were ready to support CDTi. Nigeria was one of the first and largest countries to implement REMO. Though all REMO surveys were reportedly undertaken according to the standard procedures as described in the REMO manual, there was no direct APOC involvement in the actual survey activities in the field during the first years. The main system of quality control was the review of the survey results by the Technical Consultative Committee (TCC) of APOC, a group of experts who technically advise the programme. Where TCC had reservations about survey results, the REMO surveys were repeated with participation of external experts. In case the results of these validation surveys deviated from the initial findings (which did happen in a few cases [69]), the original data were replaced by the validation data. During the first years, there was incomplete standardization of reporting: all REMO teams did report for each surveyed village the critical survey data, i.e. the village coordinates and the percentage of examined adults with nodules, but other information such as the date of survey or the number of adults examined were not always included in the reports to APOC. Furthermore, during the first years handheld GPS receivers were not yet widely available and village coordinates were often obtained using local maps. However, after this first period, all REMO surveys were implemented using handheld GPS receivers provided by APOC and with the participation of external experts to ensure standardized quality control and reporting.

### Analysis of REMO data

The analysis of the REMO data was undertaken using two analytical approaches: an expert analysis using the original REMO analytical methodology for which the results are reported in this article, and a geostatistical analysis which is described in the companion article [76].

#### Geographic information system (GIS)

All relevant geographic information was processed in a geographic information system. Initially Atlas GIS was used but since 2010 all geographic information was processed and analysed using ArcGIS 10 (ESRI Inc., Redlands, USA) including the analysis reported in his article.

The geographic information used for the analysis included:

– National and administrative boundaries, rivers and lakes, national parks, main roads, villages and urban settlements (source WHO HealthMapper http://health-mapper-release-5.software.informer.com).

– Topography and relief (source ESRI http://services.arcgisonline.com/ArcGIS/rest/services/World_Shaded_Relief/MapServer)

– Population density at 30 arc seconds resolution (source LandScan http://www.ornl.gov/sci/landscan/index.shtml)

– Areas that are unsuitable for onchocerciasis as defined during the first REMO phase (see above)

– Geographic coordinates of all surveyed villages and for each surveyed village the percentage of examined persons who had palpable nodules, referred to as the “prevalence of nodules” or “nodule prevalence” in this article.

#### Expert analysis

The original analytical approach based on expert analysis is described in detail in the REMO manual and the Guidelines for Analysis of REMO data [77,78]. Using the GIS a group of onchocerciasis experts reviewed and interpreted the REMO survey data while taking the original river-based sampling into account. The boundaries of the biogeographic zones and river basins that were used in the sampling were displayed on a map, together with spatial information on rivers and their affluents, lakes, national parks, elevation, landscape, population and administrative boundaries. For each surveyed village, the nodule prevalence was displayed as a pie chart at the location defined by its GPS coordinates. Using these maps, the experts tried to delineate the areas where the prevalence of nodules is greater than 20%, and where ivermectin treatment is therefore indicated (High Risk areas), and the areas where the prevalence of nodules is below the 20% threshold, including areas where the prevalence of nodules was zero, and where ivermectin treatment is not needed or not a priority (Low Risk areas).

**Figure 2:**
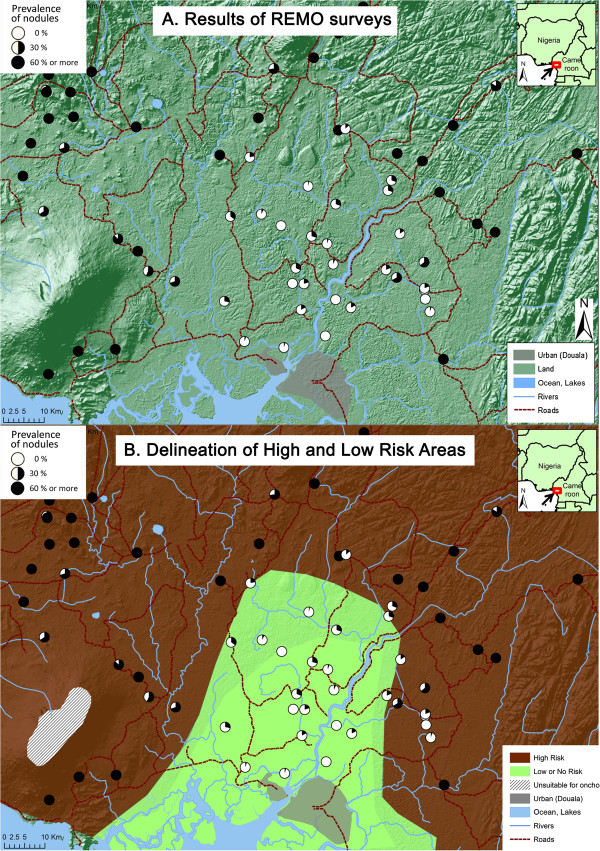
**Expert analysis of REMO data: examples for Litoral and South-West provinces in Cameroon.** Panel **A**: Results of REMO surveys. Panel **B**: Delineation of high and low risk areas.

Figure 2 gives an example of the expert analysis process for a surveyed area north of the town of Douala in Cameroon. The landscape in this area is very variable. Just around Douala there is a river delta between 5 m and 30 m above sea level. This low level area is surrounded by hills between 250 and 500 m high, a few mountain peaks in the East and North up to 1000 to 2000 m high, and Mount Cameroon in the south-east which peaks at 4040 m above sea level and were the upper reaches of the mountain are unsuitable for onchocerciasis vectors because of the high altitude. A large number of REMO surveys have been done in this area, showing very high levels of onchocerciasis endemicity in the hilly areas where most surveyed villages had a nodule prevalence > 60%, and very low endemicity in the lower reaches of the river delta. The likely explanation for this pattern is that in the hilly and mountainous areas there are many streams with rapidly flowing water that provide ample breeding sites for *Simulium* vectors, while there are no good conditions for vector breeding in the more slowly flowing waters in the low level river delta. The locally available entomological data supported this explanation. Having arrived at this basic understanding of the local epidemiology of onchocerciasis, the experts proceeded with classifying hilly areas with high nodule prevalence data as high risk areas, and the river delta area just north of Douala where the prevalence of nodules ranged between 0% and 17% as a low risk area. The boundary between these two risk areas was then drawn taking the geographic information on rivers, altitude and nodule prevalence into account. Where the nodule prevalence data were mixed, e.g. to the east where there is a borderline area with three surveyed villages with a nodule prevalence > 40% and four villages with a prevalence between 0% and 10%, the area was classified as high risk given the presence of several villages with a high prevalence of nodules.

A similar process was applied in the analysis of the REMO survey data for all other surveyed areas in the APOC countries. The classifications of all these surveyed areas was reviewed in detail in March 2010 by an APOC panel of 10 onchocerciasis epidemiologists and entomologists who refined some of the classifications and endorsed the overall map. A final round of review was undertaken in January 2013 by the staff of the epidemiological evaluation unit of APOC following some additional classifications and refinements, notably for Ethiopia where another round of REMO surveys were undertaken in 86 villages in 2012.

### Estimation of the population at high risk

The population at high risk is here defined as the rural population living in the high risk areas identified in the expert analysis. Onchocerciasis is not normally transmitted in urban settlements and urban populations that are not engaged in rural activities are excluded from estimates of the population at high risk. This exclusion criterion is not just a function of population size but depends on an assessment of the degree of urbanisation and the corresponding lack of exposure to the vector. Based on local assessments, each CDTi project decides which communities should be classified as urban and not at risk of onchocerciasis. We have, therefore, used an estimate of the density of the population at risk that is based on reported data for the target population for ivermectin treatment in ongoing CDTi projects. For each country the average population density in onchocerciasis endemic areas was estimated by dividing the reported target population in CDTi projects by the surface area for these projects. The total population in high risk areas was subsequently obtained by multiplying for each country the high risk surface area and the average population density for onchocerciasis endemic areas in that country.

### Ethical considerations

REMO is the first step in national planning for onchocerciasis control and in each APOC country it was undertaken under the auspices and ethical responsibility of the Ministry of Health. All REMO surveys were undertaken according to the WHO recommended methodology that was reviewed and approved by the Technical Consultative Committee of APOC and the Ministries of Health of the APOC countries. All REMO implementation plans were reviewed and cleared by the respective Ministry of Health and TCC. A major ethical consideration was the need to have reliable information on the geographic distribution of onchocerciasis to ensure that all populations at high risk of onchocerciasis would receive ivermectin treatment. In each village, the importance of the survey for possible future ivermectin treatment in the village was explained and village members were encouraged to participate in the examination. Participation was not compulsory and those who were requested to participate but decided not to come to the examination point, were not followed up. The examination itself was a simple non-invasive examination undertaken in a secluded location in the village.

## Results

### Unsuitable and uninhabited areas

The first step in the implementation of REMO in each country involved the identification of possible areas that are unsuitable for onchocerciasis transmission, and where, therefore, no further surveys were required. Large unsuitable areas were identified in eight countries: Central African Republic, Chad, Congo, Ethiopia, Kenya, Sudan, South Sudan and Tanzania. They included vast arid areas in Chad, Sudan and Ethiopia and swamps in Congo and South Sudan where the lack of fast flowing water made these areas unsuitable for Simulium breeding. In Sudan the area along the Blue Nile was also classified as unsuitable for onchocerciasis based on reports of previous surveys that found no cases of human onchocerciasis in the area even though the vector was sometimes present at very low densities [31]. Most of Kenya was classified as unsuitable on the basis of extensive previous entomological work that had shown that the only vector in the country was *S.naevi* and that its distribution was restricted to Nyanza province in the West of the country [25,39]. The dry central plateau of Tanzania was classified as being too dry to be suitable for Simulium breeding.

National parks were also excluded from further analysis. Population density was not used as an exclusion criterion. However, for areas where, according to the LandScan database, the population density is extremely low (<1 person per km^2^) this information is displayed on the maps.

### REMO surveys

The database used for the analysis consisted of REMO data for 14,341 villages where pre-control surveys were undertaken between 1995 and 2012. In addition, pre-control data were made available to APOC for 132 villages from Nigeria (101 villages), Cameroon (18 villages) and Tanzania (13 villages) where skin snip surveys had been done. For the purpose of analysis, the prevalence of microfilaria for these 132 villages was converted into the prevalence of nodules using the relationship between these two indicators described in a recent publication [79]. The final database consisted of survey data for 14,473 villages in which more than half a million people were examined for onchocercal nodules (Table 1).

**Table 1:** Summary of the REMO surveys undertaken in the 20 APOC countries

**Country**	**Villages surveyed**	**# of persons examined**		**Examined persons with palpable nodules**		**Prevalence of nodules per village (%)**		
		**Total**	**per village**	**Total**	**Percentage**	**Minimum**	**Median**	**Maximum**
Angola	763	25,758	34	2,491	9.7	0.0	5.3	63.3
Burundi	150	6,053	40	501	8.3	0.0	3.3	83.3
Cameroun	817	30,179	37	8,625	28.6	0.0	20.0	100.0
Central African Republic	1,078	34,984	32	15,952	45.6	0.0	50.0	100.0
Chad	483	15,795	33	2,348	14.9	0.0	6.7	96.7
Congo	384	13,853	36	1,352	9.8	0.0	3.3	71.4
Democratic Republic of Congo	4,389	170,799	39	53,501	31.3	0.0	23.3	100.0
Equatorial Guinea	209	7,751	37	1,527	19.7	0.0	11.8	73.3
Ethiopia	885	30,355	34	5,458	18.0	0.0	14.6	81.5
Gabon	59	1,633	28	29	1.8	0.0	0.0	11.8
Kenya	94	3,822	41	8	0.2	0.0	0.0	4.8
Liberia	89	4,208	47	798	19.0	0.0	20.0	35.0
Malawi	291	13,122	45	543	4.1	0.0	0.0	36.0
Mozambique	289	10,325	36	99	1.0	0.0	0.0	16.2
Nigeria	2,716	127,459	47	21,165	16.6	0.0	12.0	96.0
Rwanda	89	3,126	35	20	0.6	0.0	0.0	6.0
South Sudan	473	16,501	35	2,211	13.4	0.0	10.0	93.3
Sudan	427	21,330	50	175	0.8	0.0	0.0	23.3
Tanzania	331	20,592	62	5,035	24.5	0.0	20.8	100.0
Uganda	457	18,723	41	4,772	25.5	0.0	20.0	100.0
Total	14,473	576,368	40	126,612	22.0	0.0	14.0	100.0

**Figure 3:**
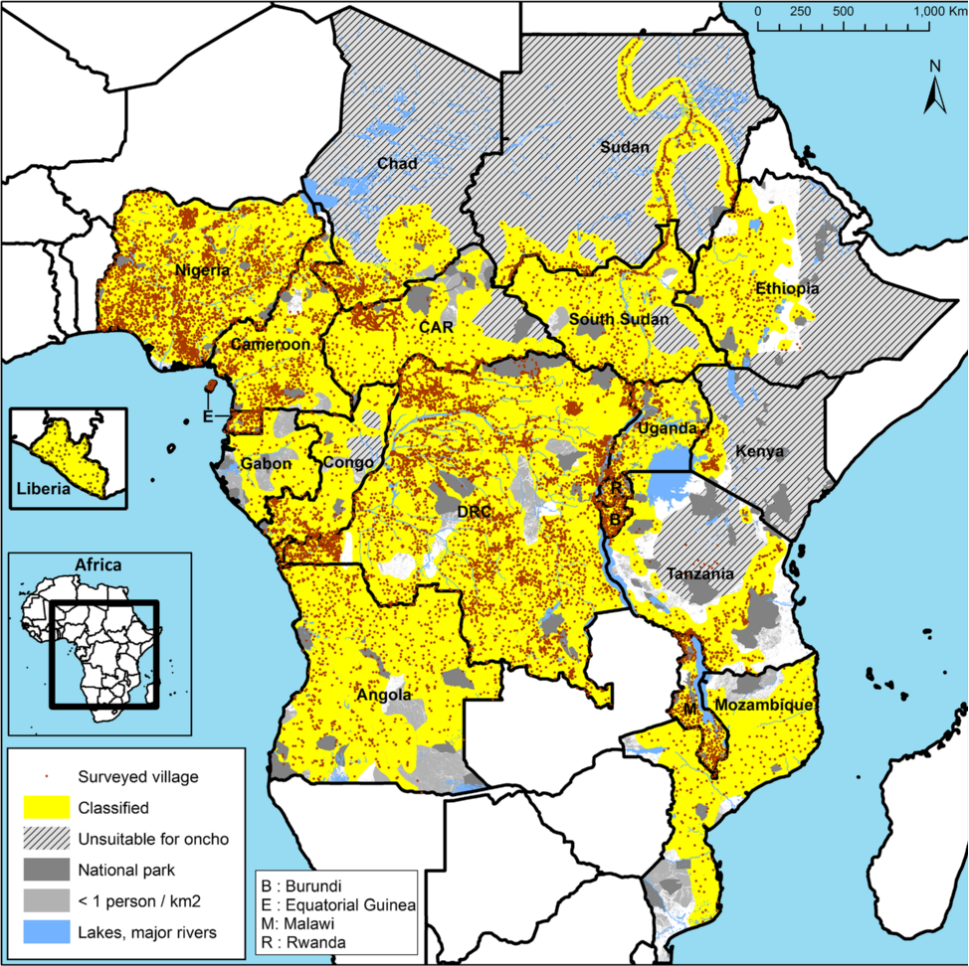
Location of the 14,473 surveyed villages in the 20 APOC countries.

Figure 3 shows the locations of the surveyed villages and of the classified area, i.e. the area that the experts classified as having high or low risk based on the survey results. The sampling density of survey villages varied between countries as the national onchocerciasis control programmes in several countries decided to survey villages at shorter distances than the recommended distance of 30 to 50 km between sample villages [77]. The reasons for these decisions included the need for very detailed onchocerciasis endemicity maps in areas that bordered zones where loiasis was highly endemic and where there was therefore a high risk of severe adverse reactions to ivermectin treatment (e.g. Bas Congo in the Democratic Republic of Congo [69]). Other reasons were highly variable relief in some areas with significant local variations in elevation (Rwanda, Burundi, Ethiopia), the desire by some national programmes to have more detailed information (e.g. Malawi), or a decision by a local CDTi project to do REMO surveys in all villages in its catchment area in order to collect a complete set of baseline data for onchocerciasis control (e.g. Ouham-Pendé Prefecture in the Central African Republic). In none of the classified areas did the average distance between neighbouring sample villages exceed the recommended distance of 30 to 50 km.

In 92% of the surveyed villages, the recommended number of 30 to 50 adults was examined for palpable nodules. In 2.2% of the villages, more than 50 adults were examined and in 5.8% of villages the number of examined adult males was less than 30. In some areas the selected villages were often too small to reach the recommended minimum of 30 adult males for examination. This was notably the case in Gabon where the threshold of 30 adults could not be reached in 62% of the selected villages and where on average 28 adults were examined per village (Table 1), and in parts of Mozambique and Rwanda where some 25% of selected villages were too small to obtain a sample of at least 30 adult males. In the original sample there were 60 very small settlements with less than 12 adult males examined and these were excluded from the analysis.

Of the total of 576,368 persons examined, 22.0% had palpable onchocercal nodules. The prevalence of palpable onchocercal nodules varied significantly between villages and countries. In all countries there were surveyed villages where the prevalence was zero. The maximum prevalence per village varied from 4.8% in Kenya to 100% in five countries (Cameroon, Central African Republic, Democratic Republic of the Congo, Tanzania and Uganda). For Gabon, Kenya, Mozambique and Rwanda the highest prevalence of nodules per village was between 4.8% and 16.2%, i.e. everywhere below the high risk threshold of 20%. The highest median prevalence of 50% was observed in the Central African Republic, followed by Cameroon, Democratic Republic of Congo, Tanzania and Uganda with a median prevalence of palpable onchocercal nodules per village between 20% and 23%.

### High risk areas

**Figure 4:**
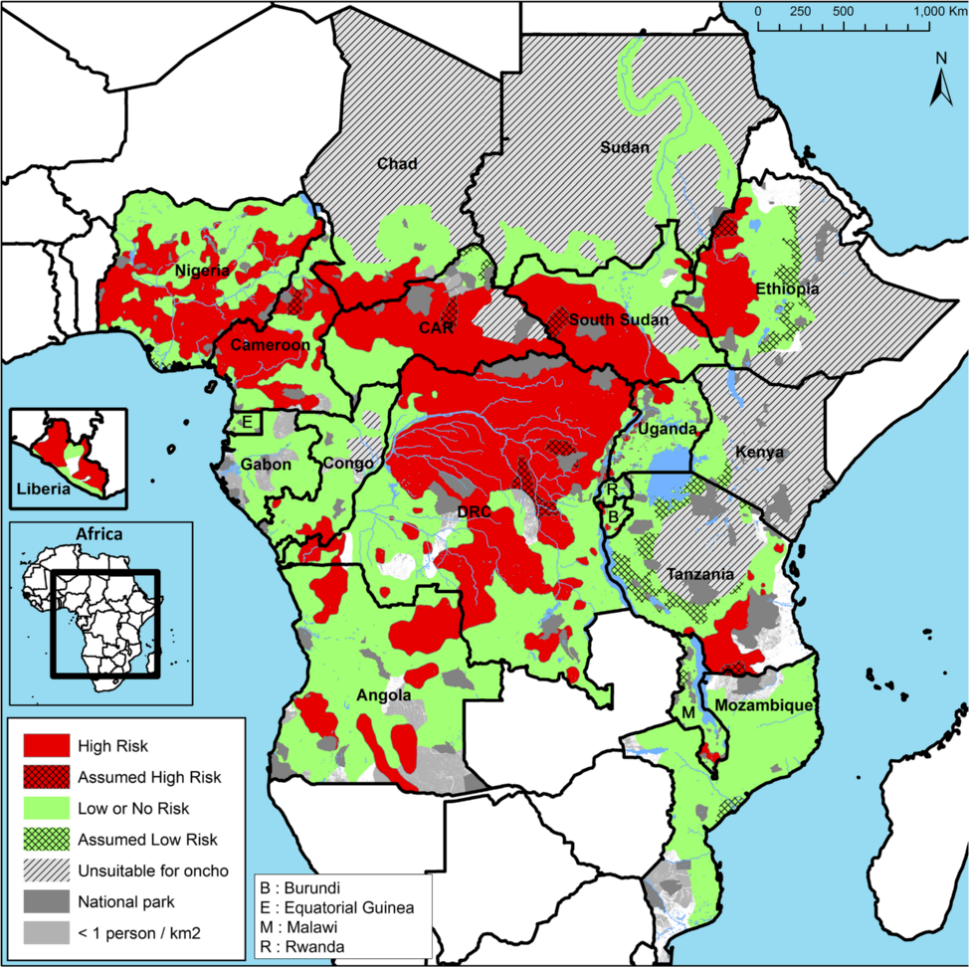
High Risk and Low Risk areas in the APOC countries – results of the expert analysis.

The main results of the expert analysis are given in Figure 4 which shows the areas classified as high risk and low risk for onchocerciasis in the 20 APOC countries. There is a vast, contiguous high risk area in the centre of Africa covering most of the Democratic Republic of Congo, South Sudan and the Central African Republic, extending in the South into Angola, in the East into Uganda and in the West into South Chad, Cameroon and Nigeria. Large high-risk areas are also found in Angola, Congo, Tanzania, Ethiopia and Liberia, and smaller but densely populated high risk areas in Malawi and Burundi. Cross-border foci are common and found in all APOC countries with high risk areas. Kenya, Rwanda, the mainland of Equatorial Guinea, as well as nearly all of Gabon, Mozambique and Sudan were classified as low risk. In the Abu Hamed focus in Sudan the prevalence of nodules was just below 20%, but in this area hyper-reactive onchodermatitis or Sowda is common, and the 20% prevalence may not be an adequate threshold for the public health importance of the disease in this focus.

There are some areas that could not be classified because no REMO surveys had been done. However, as shown in the map, many of these unclassified areas had a very low population density of <1 person per km^2^. Only for a few more densely populated areas there is no survey data available, notably in Tanzania, Angola and the Democratic Republic of Congo. For some unsurveyed but populated areas the surrounding zones were all classified as high risk while the environment was similar in terms of climate, hydrology, vegetation and altitude. Such areas were therefore classified as “assumed high risk” pending future validation by additional surveys where still considered necessary. Likewise, some unsurveyed populated areas were classified as “assumed low risk” if all the surrounding areas were consistently classified as low risk and the environment was similar between those areas.

The total surface of the areas classified as high risk is 3.3 million km^2^ (Table 2), representing 24% of the surface of the 20 APOC countries. In Cameroon, the Central African Republic, the Democratic Republic of Congo and Liberia more than half the surface of the country was classified as high risk for onchocerciasis, while in South Sudan and Nigeria the high risk area represents 49% and 43% of the country respectively.

**Table 2:** High risk area and population by country

**Country**	Surface* (1000 km2)	Population** (1000)	**Population per km2 in CDTi areas**	**High risk area**			
				**Surface (1000 km2)**	**% of country surface**	**Population (1000)**	**% of country population**
Angola	1,247	19,082	3.8	262.3	21.0%	985	5.2%
Burundi	28	8,383	341.0	3.4	12.3%	1,166	13.9%
Cameroon	475	19,599	24.2	249.5	52.5%	6,027	30.8%
CAR	623	4,401	5.2	328.1	52.7%	1,693	38.5%
Chad	1,284	11,227	21.6	93.7	7.3%	2,024	18.0%
Congo	342	4,043	34.8	20.9	6.1%	727	18.0%
DRC	2,345	65,966	22.1	1,247.3	53.2%	27,573	41.8%
Eq. Guinea	28	700	22.0	1.1	3.8%	87	12.4%
Ethiopia	1,104	82,950	46.7	177.1	16.0%	8,271	10.0%
Gabon†	268	1,505	3.8	0.7	0.3%	3	0.2%
Kenya	584	40,513	NA	0.0	0.0%	0	0.0%
Liberia	96	3,994	30.2	63.1	65.5%	1,904	47.7%
Malawi	118	14,901	237.4	7.2	6.1%	1,713	11.5%
Mozambique†	799	23,391	18.0	2.6	0.3%	46	0.2%
Nigeria	924	158,423	65.3	400.2	43.3%	26,120	16.5%
Rwanda	25	10,624	NA	0.0	0.0%	0	0.0%
South Sudan	644	10,693	13.8	312.5	48.5%	4,313	40.3%
Sudan	1,861	35,048	14.6	0.6	0.0%	9	0.0%
Tanzania	947	44,841	19.4	91.9	9.7%	1,783	4.0%
Uganda	242	33,425	56.4	25.5	10.6%	1,437	4.3%
Total	13,986	593,709	27.1	3,288	23.5%	85,881	14.5%

*Source: http://wdi.worldbank.org/table/1.1.

**Source: http://esa.un.org/wpp/Excel-Data/population.htm.

†Population estimated using LandScan data for the high risk areas in the country.

In Gabon and Mozambique only 0.3% of the surface was classified as a high risk area. In both cases, it concerned a narrow border area where no REMO surveys were done within the country itself but where the presence of a large endemic focus on the other side of the border in the neighbouring country suggests that there may be also some hyperendemic villages on the banks of the border river within Gabon and Mozambique. Except for those narrow border areas, the REMO data for these two countries do not indicate the presence of any high risk areas.

### Population of high risk areas

The total population of the high risk areas is estimated at 86 million for the year 2011, corresponding to some 15% of the total population of the 20 APOC countries. Most of the high risk area population comes from Nigeria (26 million) and the Democratic Republic of Congo (28 million). In Cameroon, the Central African Republic, the Democratic Republic of Congo and Liberia, the population of high risk areas represents some 31 to 48% of the total population of the country.

## Discussion

APOC’s first major task was to determine where onchocerciasis was a public health problem and where ivermectin treatment should therefore be targeted. It was a daunting challenge because of the vast area in the 20 APOC countries where onchocerciasis was potentially endemic. Only by using the rapid assessment method REMO, APOC was able to map the high risk areas within a limited period of time so that CDTi projects could be launched without significant delays. However, even though REMO is a rapid assessment method, its implementation remained a major effort in which more than 14,000 villages were surveyed and over half a million people examined for the presence of palpable nodules. With the exception of a few small areas, REMO has now been completed in all APOC countries. A comprehensive map of the geographic distribution of onchocerciasis in the 20 APOC countries has been produced which clearly delineates the high risk areas where ivermectin treatment is a priority.

High risk areas were identified in all APOC countries except Kenya and Rwanda. They ranged from small, isolated onchocerciasis foci (e.g. the island of Bioko in Equatorial Guinea) to a vast contiguous endemic area of more than 2 million km^2^ that runs across seven countries. In five countries (Cameroon, Central African Republic, the Democratic Republic of Congo, Liberia and South Sudan) the high risk area covers more than 49% of the total surface area of the country, and between 31% and 49% of the total population. An estimated 86 million people (2011 estimate) live in high risk areas in the 18 APOC countries, with the majority living in the Democratic Republic of Congo (28 million) and Nigeria (26 million).

In Kenya and Rwanda there were no areas classified as high risk. In Kenya, only 8 persons out of 3822 examined (0.2%) had palpable nodules and given that nodule palpation is not 100% specific [79], these results appear to confirm the previous conclusion that onchocerciasis has been eliminated from Kenya [1]. In Rwanda the overall nodule rate was also extremely low, suggesting that the country is non-endemic for onchocerciasis.

The high risk maps confirmed that the REMO surveys were very necessary. Several of the newly identified high risk areas were previously not even reported to have onchocerciasis [1], e.g. five of the six high-risk areas in Angola, the high risk areas in the north-west and south-east of the Democratic Republic of Congo, the high risk areas in the extreme north of Nigeria and in the extreme south of Cameroon, and the extent of the high-risk zone in south Tanzania. Furthermore, for most areas that were previously thought to be endemic for onchocerciasis, the available data were insufficient to assess the level of endemicity and thus the importance of the disease as a public health problem. The REMO data clarified where the disease was a severe public health problem and where ivermectin treatment was a priority, thus providing the critical information needed for launching CDTi in the APOC countries.

REMO has limitations that should be taken into account when interpreting the risk maps. The first relates to the use of onchocercal nodule palpation as an indicator of onchocerciasis infection. Onchocercal nodule palpation is subject to observer variation, and although APOC has provided standard training in nodule palpation to all examiners, this is unlikely to have eliminated all observer variation between the many examiners in the different countries. Secondly, the prevalence of nodules is only a proxy for the risk of onchocercal disease, and the prevalence of 20% palpable nodules only an approximate threshold for the endemicity level where the risk of severe onchocercal complications becomes significant. The differentiation between high and low risk areas is based on previous work which has shown that onchocercal blindness is rare when the prevalence of microfilaria is below 35% (corresponding to a nodule prevalence of about 20%), but that the risk of blindness increases with the prevalence of microfilaria, and becomes very severe when the prevalence of microfilaria exceeds 60% (or 40% nodule prevalence). Given that there is no sharp cut off point for the risk of onchocercal disease and taking into account the small average sample size of 40 adults examined per village, the experts tended to be cautious when the prevalence of nodules was around 20% and classified such areas as requiring ivermectin treatment.

There was significant variation in village sampling density between countries. Some countries applied the minimum of at least one sample village every 30-50 km along the main rivers and affluents, as recommended in the REMO manual. Other countries and some CDTi projects selected REMO villages at much shorter distances. However, in none of the surveyed areas did the average distance between neighbouring sample villages exceed the recommended distance of 30 to 50 km. There was limited variation in the number of adults examined per village, and in 92% of the sample villages the recommended number of 30 to 50 adults was examined for palpable nodules. Only in 5.8% of the villages was the number of examined adults less than 30, and the reason was usually that the selected villages were too small to reach the required number of 30 adults who were willing to participate in the examination. This was notably the case for sample villages throughout Gabon and in parts of Mozambique and Rwanda.

Although the process and criteria used in the expert analysis are defined in the REMO manual, there remains a substantial subjective element in the classification of risk areas using the expert approach. For many river basins the risk pattern was very clear, e.g. when the prevalence for all surveyed villages was greater than 20% and the whole basin was classified as a high risk area, or when all prevalence rates were near zero and the whole basin is classified as low risk. However, for other river basins, the results were more variable making it sometimes very difficult to draw the boundary between areas where the prevalence was above or below the 20% threshold. In the original REMO guidelines, it was proposed to undertake additional surveys in areas where the epidemiological pattern was not sufficiently clear in order to improve the classification. However, additional surveys did not prove a solution in borderline areas where the prevalence of onchocercal nodules fluctuates around 20%. The experts tended to classify such areas as high risk to ensure that subpopulations at high risk onchocerciasis were not denied ivermectin treatment. Hence, the expert analysis had a tendency to overestimate high risk areas.

The reliability of the estimates of the population at high risk depends on the population density estimates used. We estimated this population density by the average population density in the CDTi projects in each country. This provides, in our opinion, the most reliable estimate of the density of the population at risk in onchocerciasis endemic areas as it excludes urban populations that are not considered to be at risk. However, these estimates are based on reported population data for CDTi projects. These reported data may not always be reliable, especially during the first years of a CDTi project when it is not yet covering all villages in the project area or during later years when community directed distributors (CDD) sometimes only report the population eligible for treatment rather than the total population in the village. In both cases the effect would have been underestimation of the population at high risk.

## Conclusions

The REMO maps have played a significant role in onchocerciasis control in the APOC countries. The clear delineation of high risk areas has led to the creation of more than 100 CDTi projects that by 2012 were covering a total population of 107 million people and treating over 80 million of them who were eligible for treatment [80]. By the year 2013, CDTi projects were covering nearly all high risk areas in the APOC countries, thus ensuring the control of the disease as a public health problem and preventing 2 million Disability Adjusted Life Years (DALYs) per year [81,82]. The main exception was the Democratic Republic of Congo where the implementation of CDTi has been delayed because of civil unrest. However, in this country the coverage of CDTi is now expanding rapidly, helped by a clear understanding of where the priority areas for treatment are located.

The large scale application of REMO has provided an evidence-based map of priority areas for ivermectin treatment for onchocerciasis in 20 African countries. The mapping has evolved in synchrony with the progression of onchocerciasis control, first providing detailed high risk maps for early uptake countries and subsequently mapping high risk areas in other APOC countries to guide their national CDTi planning. The final result is a detailed epidemiological map of direct operational relevance for onchocerciasis control that is unique among neglected tropical diseases. This rapid, evidence-based delineation of target areas for intervention is often quoted as one of the main reasons for the success of the African Programme for Onchocerciasis Control [81,83].

## Competing interests

The authors declare that they have no competing interests.

## Authors’ contributions

MN and JHFR were involved in the design of REMO. MN, AT, HZ and JHFR planned the implementation of REMO. MN, AT, PE and BEBN supervised and implemented the REMO surveys. HZ, MN and AT were responsible for data processing. MN and JHFR coordinated the expert analysis. MN, HZ, AT and JHFR did the final analysis for the article. JHFR drafted the manuscript and all authors contributed to and approved the final manuscript.
